# Laser Fluorescence Illuminates the Soft Tissue and Life Habits of the Early Cretaceous Bird *Confuciusornis*

**DOI:** 10.1371/journal.pone.0167284

**Published:** 2016-12-14

**Authors:** Amanda R. Falk, Thomas G. Kaye, Zhonghe Zhou, David A. Burnham

**Affiliations:** 1 Centre College, Department of Biology, Danville, KY, United States of America; 2 Burke Museum of History and Culture, Seattle, WA, United States of America; 3 Key Laboratory of Vertebrate Evolution and Human Origins, Institute of Vertebrate Paleontology and Paleoanthropology, Beijing, China; 4 University of Kansas Natural History Museum and Biodiversity Institute, Lawrence, KS United States of America; University of Akron, UNITED STATES

## Abstract

In this paper we report the discovery of non-plumage soft tissues in *Confuciusornis*, a basal beaked bird from the Early Cretaceous Jehol Biota in northeastern China. Various soft tissues are visualized and interpreted through the use of laser-stimulated fluorescence, providing much novel anatomical information about this early bird, specifically reticulate scales covering the feet, and the well-developed and robust pro- and postpatagium. We also include a direct comparison between the forelimb soft tissues of *Confuciusornis* and modern avian patagia. Furthermore, apparently large, fleshy phalangeal pads are preserved on the feet. The reticulate scales, robust phalangeal pads as well as the highly recurved pedal claws strongly support *Confuciusornis* as an arboreal bird. Reticulate scales are more rounded than scutate scales and do not overlap, thus allowing for more flexibility in the toe. The extent of the pro- and postpatagium and the robust primary feather rachises are evidence that *Confuciusornis* was capable of powered flight, contrary to previous reports suggesting otherwise. A unique avian wing shape is also reconstructed based on plumage preserved. These soft tissues combined indicate an arboreal bird with the capacity for short-term (non-migratory) flight, and suggest that, although primitive, *Confuciusornis* already possessed many relatively advanced avian anatomical characteristics.

## Introduction

With the recent discoveries of magnificently preserved fossils from the Early Cretaceous Konservat-Lagerstätten (Jehol Biota) of northeastern China—many of which exhibit exceptional soft-tissue preservation, including plumage—a rare opportunity to study the soft-tissue anatomy of fossil birds arises. *Confuciusornis* has been well studied with regards to its osteology (e.g., [1–4]) and some portions of its plumage. Much about the plumage, however, has yet to be adequately described, and much of the current research has focused on the primary flight feathers and the enigmatic, long, paired tail feathers [5–7]. The paired tail feathers of *Confuciusornis* were suggested to represent sexual dimorphism [1] and their exact morphology has been the subject of debate (e.g., [7]). The primary feathers of *Confuciusornis* were interpreted as thin and weak, and therefore unable to support flapping flight [5]. Furthermore, the primary feathers of *Confuciusornis* were much longer relative to the arm bones than what is found in modern birds, and previous studies suggested that *Confuciusornis* possessed a long and pointed, fast-flying type of wing morphology [6]. Although there have been studies refuting the suggestion of the thin and weak primary rachises [8,9], some studies still maintain that the feathers were too weak for flapping flight (e.g., [6]).

Modern birds have unique soft tissues that relate to their flight capability and preferred life habits. Modern birds possess both a propatagium and a postpatagium [10], which are expandable membranes on both the front and the back of the wing. The propatagium has been suggested as the important lift-producing structure in the proximal portion of the avian wing [11]. The function of the postpatagium in birds is less well understood, but is the main area of support beyond the osteological insertion of the inferior umbilicus of the main flight feathers, the primaries and secondaries. Strong tendons connect feather calami within the postpatagium, especially in areas where the strongest external forces act upon the feathers [12]. Furthermore, scales on the feet and tarsi can potentially be linked to life habit and evolutionary patterns [13, 14]. Unfortunately, these delicate soft tissue structures are rarely preserved in the vast majority of fossil birds; however, many specimens from the Lower Cretaceous Jehol Group are preserved with spectacular plumage and other soft tissues.

Laser-stimulated fluorescence (LSF) is a process widely used in various subfields of biology today (e.g., use of green fluorescent protein, [15]), and to microfossils (e.g. [16–18]), but has only recently been successfully applied to macrofossils [19]. LSF is a highly successful and versatile method that can be used to identify potentially modified fossils, highlight hidden structures, and, as this study shows, fluoresce soft tissues otherwise invisible under white light.

## Materials and Methods

Laser-stimulated fluorescence uses the high flux of a laser to induce fluorescence in minerals that comprise fossil bone, matrix and other compounds. In its simplest form, an intense laser beam is scanned across the specimen in a dark room while a camera records a time exposure through a laser blocking filter. In this way only the fluorescence coming from the specimen is recorded. See Kay et al. [19] for further details on the methodology of laser-stimulated fluorescence.

For this study a pair of blue 447 nanometer (nm) lasers of 300 and 400 milliwatts respectively were used. The handheld portable unit which was brought to the site was used standalone without mains power, the other a standard laboratory style laser. The handheld configuration limits the laser on-time to 5 minutes. The beam was slightly defocused through a Thorlabs ED1-S50-MD diffuser with a 90% transmission rate. A Nikon D60 SLR camera was configured on a tripod in bulb mode to facilitate time exposures. ISO was set to 800 and typical exposure times were in the range of 1.6-1/1.6 seconds. A MidOpt Light Yellow LP 470 ‘longpass’ filter with a >90% passband above 535 nm was mounted to the front of a Nikkor 18–55 mm standard lens and a Nikkor 85mm macro lens to block the laser flux and only allow imaging of the fluorescence.

**Fig 1:**
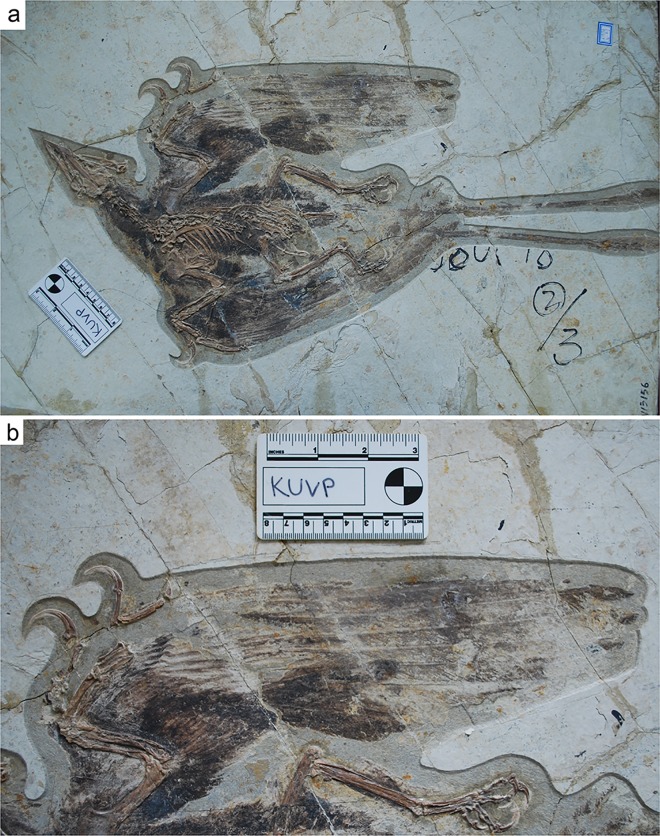
Photograph of IVPP V13156. (A) Full specimen. (B) Close up of right wing. Note the shortened tenth (outermost) primary.

Aspect ratio for a reconstructed wing of *Confuciusornis* was calculated following Pennycuick [20]. A reconstruction of the wing of *Confuciusornis* was created using measurements from IVPP V13156 and imported into Adobe Illustrator CS4. Within Illustrator “view grid” was selected, allowing for wing span and wing area to be calculated. The full wing span measured by using semi-span, using approximate body width measured directly from the body IVPP V13156, as the torso of the specimen is preserved ventral-side down, and there is a clear body outline (Fig 1). After the semi-span was calculated, the measurement was multiplied by two to obtain a wing span as specified by [20]. Wing area was generated by counting the number of grid squares that were completely within the reconstruction (full squares), squares that were only half filled by a portion of the reconstruction (half-squares), and squares that were either more than half or less than half filled by a portion of the reconstruction. These squares were added to get an approximate partial wing area as shown in [20] The length of each square was calculated as 3 cm. Root chord was added to the semi-span following [20] to calculate the total wing area for one wing; this number was then multiplied by two to obtain the full wing area. Using these measurements, aspect ratio was calculated using the formula used by Pennycuick [20].

## Description

**Fig 2:**
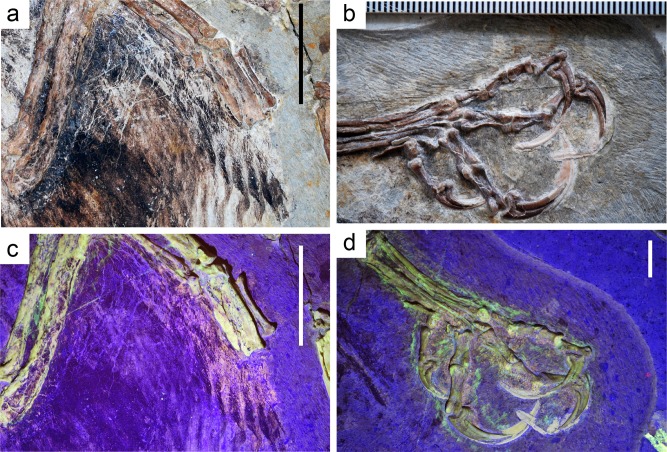
Comparing photographs taken under white light and laser fluorescence. (A) Wing of IVPP V13156 under white light. Scale bar = 2 cm. (B) Right foot of IVPP V13156 under white light. Scale in mm. (C) Wing of IVPP V13156 under laser fluorescence. Scale bar = 2 cm. (D) Right foot of IVPP V13156 under laser fluorescence. Scale bar = 1 cm.

Two specimens of *Confuciusornis* from the Institute of Vertebrate Paleontology and Paleoanthropology (IVPP) in Beijing were especially useful for this study; IVPP V13168, and IVPP V13156 (Fig 1). Under laser fluorescence (see materials and methods), soft tissues including scales, toe pads, skin, and muscle are highlighted around the feet, wings, pygostyle and hindlimb. These structures were not visible under white light alone (Fig 2), and ultraviolet (UV) light did not produce the clarity of image seen with the laser fluorescence. The location, pattern, and shape of these tissues closely match soft tissue structures seen in modern birds, especially phalangeal toe pads and metatarsal pads on the feet, and pre- and postpatagium on the wings.

**Fig 3:**
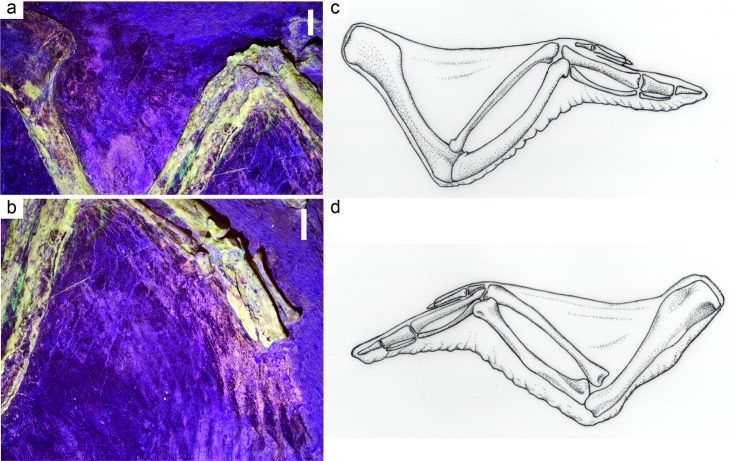
Photographs of soft tissue under laser fluorescence from IVPP V13156. (A) Wing showing propatagium. Scale bar = 5 mm. (B) Wing showing postpatagium. Scale bar = 5 mm. (C) Line drawing of a modern *Gallus gallus* wing showing outline of patagium in relation to the bones of the forelimb, dorsal view. (D) Line drawing of a modern *Gallus gallus* wing showing outline of patagium in relation to the bones of the forelimb, ventral view. (C) and (D) by Elizabeth Myers.

**Fig 4:**
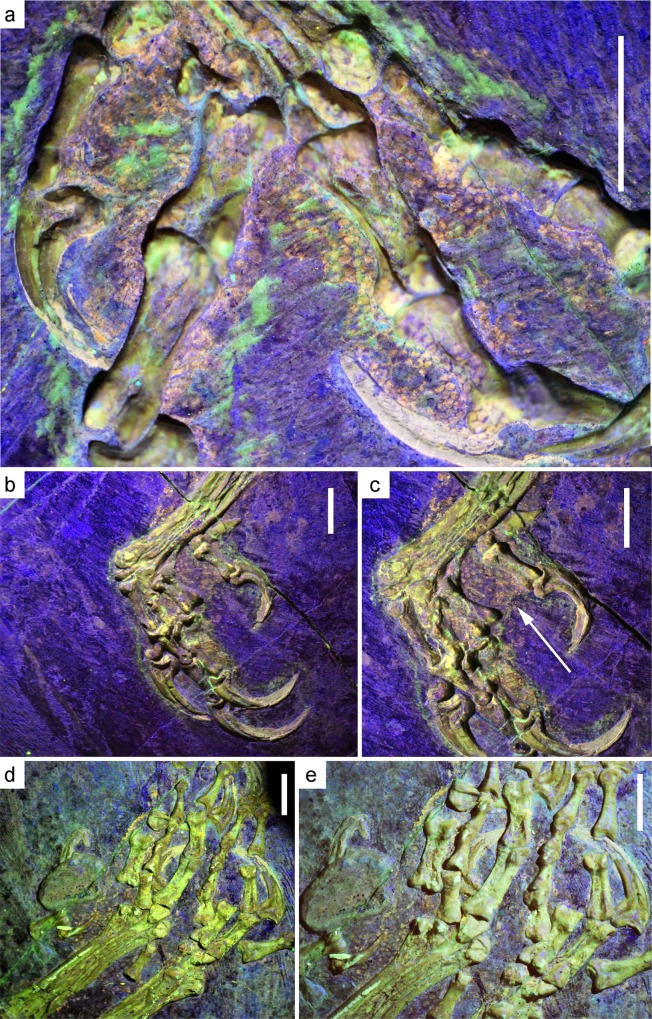
Photographs of the feet of IVPP V13156 and IVPP V13168. (A) Closeup of right foot, yellow longpass. Scale bar = 7 mm. Note the yellow-orange scales concentrated around the phalanx, not the joint (B) Left foot, yellow longpass. Scale bar = 7 mm (C) Close up of left foot, yellow longpass. Scale bar = 7 mm. Note the fleshy pad with scales indicated by the arrow. (D) IVPP V13168, yellow longpass. Scale bar = 5 mm (E) Close up of IVPP V13168, yellow longpass. Scale bar = 5 mm.

Both propatagium and postpatagium are visible on IVPP V13156 as pinkish halos around bone that also produces yellow fluorescence (photographs unprocessed) (Fig 3A and 3B). The halo is wider proximally and narrows distally down to the joint. There are scales and pads seen preserved on the feet for the first time (Fig 4), which are positioned ventral-side up. Scales are present on both the matrix, and on the bone of the phalanges and the tarsometatarsus. Modern birds show two types of scales; reticulate and scutate. Reticulate scales are smaller, rounder, and do not overlap. Scutate scales are larger, more rectangular, and overlap. The scales seen on *Confuciusornis* are entirely reticulate, and there are no scutate scales visible.

The wing of IVPP V13156 is slightly extended, and so an accurate reconstruction of the propatagium is easily produced. The propatagium of *Confuciusornis* was extensive, stretching from shoulder to wrist as in modern birds. The brightest part of the halo interpreted to be propatagia extends ~11–12 mm from the radius, however the exact width is variable. The halo is fainter further away from the bones, but extends to ~20 mm away from the radius in the same location as the broadest portion of the brighter region. Under white light, a black halo of organic tissue extends ~36 mm from the radius. The thickness of the propatagium is indeterminate; however, the amount of organic material, and the clarity of the halo under laser fluorescence and UV light, suggests that the propatagium was not a thin membrane but a substantial structure as found in modern birds. The propatgium seen on *Confuciusornis* is similar in its outline to the propatagium found in modern birds (Fig 3C and 3D).

The postpatagium covering the ulna of IVPP V13156 is estimated to be between 5–6 mm from the caudal margin of the ulna to the edge of the postpatagium. The postpatagium on the hand is ~10 mm as measured from the caudal margin of the carpometacarpus and phalanx to the edge of the postpatagium. The extent of the postpatagium appears to be consistent with that of modern avifauna (e.g., *Gallus gallus*, *Bubo virginianus* [21]) and is similarly distributed (Fig 3D).

**Fig 5:**
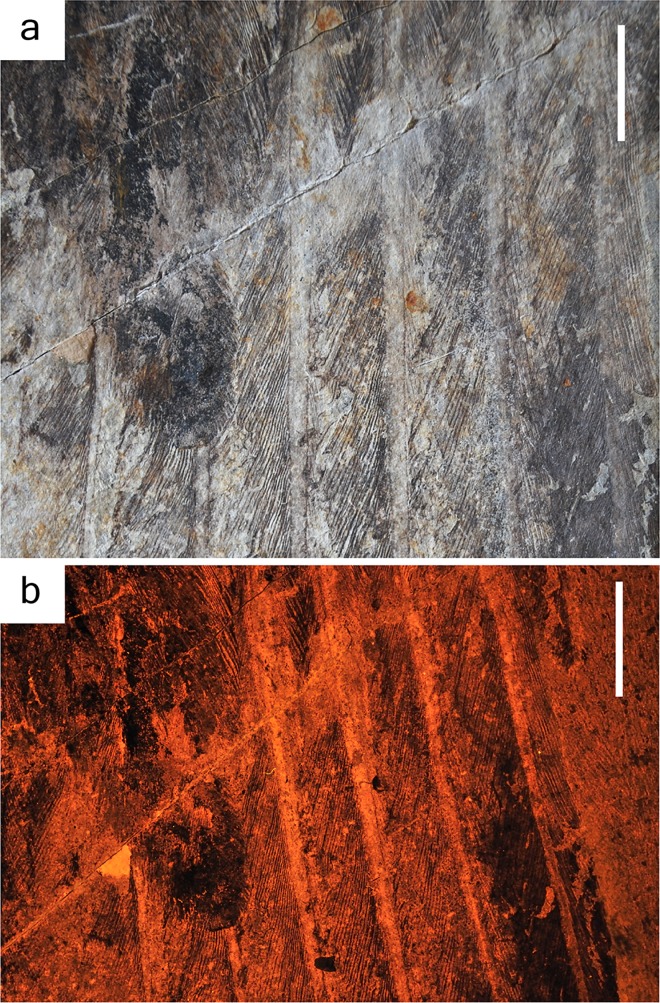
Photographs of the primary rachises of *Confucuisornis*, IVPP V13156. (A) Rachises under white light. (B) Rachises under laser fluorescence using an orange longpass filter. Scale = 1 cm.

The primary feather morphology and wing shape of specimens IVPP V13156 and V13168 are different than previously reported for *Confuciusornis*. The rachises of specimen IVPP V13156 are robust (contra to [5; 8,9]), with the thickest measuring >1.5 mm (Fig 5), and are clearly visible under both white light and laser fluorescence. The rachis itself does not fluoresce; as explained by Kaye et al. [19], the matrix behind the feathers fluoresces, which causes a “backlit” phenomenon, highlighting detail. This suggests that no organics are preserved in the rachis itself.

**Fig 6:**
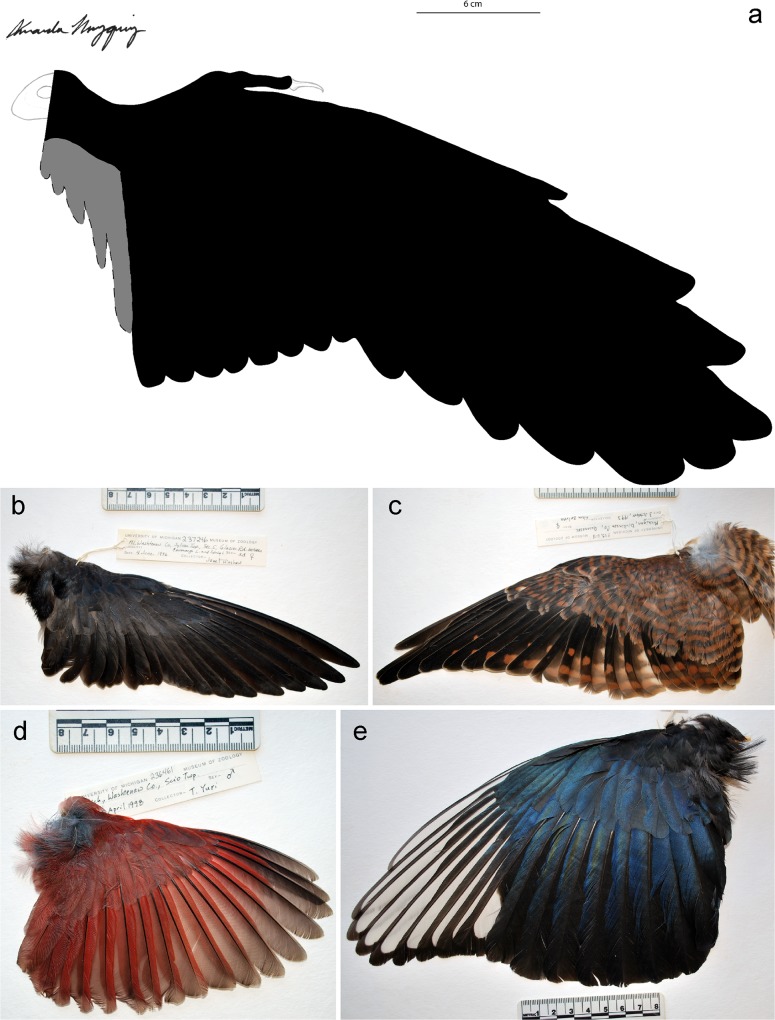
Comparison of the wing of *Confuciusornis* with the wings of modern birds. (A) *Confuciusornis*, reconstruction, dorsal view. Grey-shaded region indicates areas where tertials would be located. (Osteological reconstruction by A. Muzquiz.) (B) Purple Martin (*Progne subis*), UMMZ 237246, dorsal view. (C) American Kestrel (*Falco sparevis*), UMMZ 233618, dorsal view. (D) Northern Cardinal (*Cardinalis cardinalis*), UMMZ 236461, dorsal view. (E) Black-billed Magpie (*Pica pica*), UMMZ 235421, dorsal view.

As previously reported (e.g., [6]) the primary feathers of *Confuciusornis* are exceptionally long in relation to the forearm, however, the relative length of each individual primary, especially the outermost primaries, is different than has been previously reported. Due to the relative length of the primaries, the wing shape is much broader and rounder than previously suggested. The 10th (outermost) primary of *Confuciusornis* is less than half the length of the 9th primary, which in turn is shorter than the 8th primary (Fig 1B). The overall length of the secondaries in comparison to the primaries is also much greater than what is seen in most fast-flying birds (Fig 6) and results in a completely novel wing shape that is far different than previous reconstructions have suggested [22]. This rounder, broader morphology is more similar to that seen in both forest-dwelling and soaring birds [23], however the wing shape of *Confuciusornis* is unlike any known modern avian wing morphology (e.g., Fig 6B–6E).

The scales of *Confuciusornis* appear to be entirely reticulate. The feet, however, are preserved ventral-side up, which may obscure any scutellate scales as they are usually found on the dorsal side of the toes and tarsometatarsus. Disarticulated reticulate scales are present around the feet of IVPP V13168. The scales are relatively spread out away from the phalanges on the right foot of IVPP V13156; however, on the left foot the scales are preserved in situ relative to the phalanges. Large phalangeal pads and small interphalangeal pads can be distinguished on both left and right feet (Fig 4A and 4B). A robust metatarsal pad is clearly present, best seen on the left foot.

## Discussion

Previous studies (e.g. [1,3]) visualized only the osteology and/or plumage of *Confuciusornis*, however our study represents a first look at other soft tissues such as scales, dermal complexes (patagia), ligaments, tendons and musculature of *Confuciusornis*. This new information indicates that *Confuciusornis* has a suite of relatively modern soft tissue structures that are more advanced than may be expected. New information about the wing shape of *Confuciusornis* also reveals more about how this enigmatic bird may have flown.

The wider, broader wing in *Confuciusornis* confirms a flight style different than previously suggested (see [6]) (Fig 6). Wide, broad wings are found in 1) birds that live in a densely vegetated area requiring high maneuverability and 2) broad-winged soaring birds such as hawks and vultures. Although there is minor separation between the tips of the primaries in IVPP V13156, the presence of slotted wing tips is difficult to determine. Slotted wing tips are typical of broad-winged soaring birds, which aid in reducing drag during soaring [24,25] and may improve stability while gliding [26]. Reconstructions of a heavily forested environment surrounding the lake(s) of the Lower Cretaceous Jehol Group (e.g., [27,28]) implies *Confuciusornis* required more maneuverability and stability than speed in flight. The aspect ratio of *Confuciusornis* is between 6.4 (including tertial feathers) and 7.7 (excluding tertial feathers), which indicates that it was not a dynamic soarer (e.g., seabirds), long-distance migrant (e.g., shorebirds), or a long-duration fast flyer (e.g., swifts), which have higher aspect ratios [29].

The pinkish halos around the forelimb of *Confuciusornis* compare favorably to the patagia of modern birds (Fig 3). The area of brighter fluorescence in the propatagia may indicate a thickened area of the skin or deformation of the soft tissues after death—the dorsal portion of the propatagia in *Gallus gallus* is notably thicker than that of the ventral region (Fig 3C and 3D). The brighter region of the halo may also represent a thickened propatagial ligament found in modern birds [21, 30]. Impressions of the soft tissues of the hand of *Archaeopteryx* have been reported [31] and compare favorably to the halos seen in *Confuciusornis* and modern avian wings. The presence of pre- and postpatagial ligaments and tendons is difficult to determine from the laser fluorescence, however, these ligaments and tendons serve crucial functions for flight in birds, including adjustment of the camber of the wing (prepatagial ligaments), control over extension of the wing [32], and spreading of the flight feathers (postpatagial tendons) [20]. The likelihood of *Confuciusornis* possessing significantly robust versions of these tendons and ligaments (as seen in other birds, e.g. [21]) seems high.

**Fig 7:**
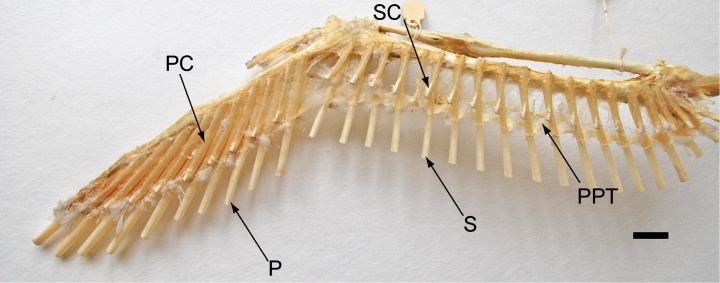
A spread-wing osteological specimen from the University of Michigan Natural History Museum. Note the insertion points of the primary feathers on the major metacarpal and digit. P = Primary, S = Secondary, PC = Primary covert feather, SC = Secondary covert feather, PPT = Postpatagial tendon.

The more postpatagium present, the more attachment area for the feather calami, and the feathers are more likely to retain a straight airfoil. Furthermore, the osteology of the wing of *Confuciusornis* supports the presence of a postpatagium. The major digit (digit 3 according to e.g. [33], digit 2 according to e.g. [3]) serves as the site of bony support and attachment for the large primary flight feathers (Fig 7). The minor digit is reduced to a vestigial stub, however the minor metacarpal is still present and robust, and often curved [10]. The minor metacarpal acts as a bony strut to support the calami of the primary feathers. This function of the minor digit in *Confuciusornis* is supported by the relative smaller size of the bone compared to the major digit (Fig 1; note thickness of the bones belonging to the major digit). Some suggest that the minor digit of *Confuciusornis* was freely mobile based on claw morphology [34], however the osteology of the hand, combined with the evidence shown via laser fluorescence, suggests that this interpretation is incorrect and that the finger was completely encased within patagium, as it is in modern birds. The manus would still be capable of clinging to tree trunks or limbs with the minor digit encased in postpatagium, similar in the way that a Hoatzin (*Opisthocomus hoazin*) uses its vestigial claws as chicks.

A recent discovery by Navalón et al. [35] displays a similar morphology, visible under white light as a dark halo surrounding the forelimb of an enantiornithine. Unlike the morphology of the enantiornithine, the propatagium of *Confuciusornis* does not include the thumb, which would have been freely mobile. This suggests that the patagial system in birds developed early in their evolution and, when available for study, may be a uniting synapomorphy. Major morphological changes including the extent and robustness of the pro- and postpatagia likely changed at key points within Aves. The more postpatagium present, the more attachment area for the feather calami, and the feathers are more likely to retain a straight airfoil.

There is evidence that the propatagium of birds contributes disproportionately to lift production between the wrist and the elbow [11], and the primary feathers generate lift from the wrist outwards. The extensive and robust propatagium in *Confuciusornis* would have generated a large amount of lift. Furthermore, a thick and deep postpatagium suggests strong tendons running parallel to the edge of the postpatagium, anchoring the flight feather calami in place [20]. The deeper and thicker the postpatagium, the stronger the attachment of the flight feathers, and the less likely the feathers are to buckle and twist. All of the above factors, along with the length and shape of the primary and secondary feathers, the robustness of the primary rachises, combined with the presence of a small but relatively extensive keel [36], strongly suggest that *Confuciusornis* did have the capability for powered flight.

Since only the underside of the foot is preserved, the interpretation of the lack of scutellate scales on the feet is limited. The dorsal side of the foot may have had scutellate scales; however, it is still embedded in the matrix. The feet of IVPP V13168 also have scales and, although they are disarticulated (Fig 4E and 4F), they are also reticulate. Many modern birds have primarily scutellate (e.g., Passeriformes) or scutellate-reticulate (e.g., Columbiformes) scaled feet, and many other groups possess only reticulate scales (e.g., plovers (Charadriiformes), falcons (Falconiformes), and parrots (Psittaciformes)) [13,37]. Reticulate scales do not overlap and are therefore more flexible than the elongate, overlapping scutate scales [14]; they are found on the ventral side of the foot in Columbiformes and many Passeriformes. Tree climbing is considered primitive for Aves [38], and a more flexible foot would have helped *Confuciusornis* cling and grasp to the tree trunks and branches of large tress. There is some evidence that dorsal reticulate scales have arisen multiple times within Aves [14], which may complicate discussions of the ancestral state.

**Fig 8:**
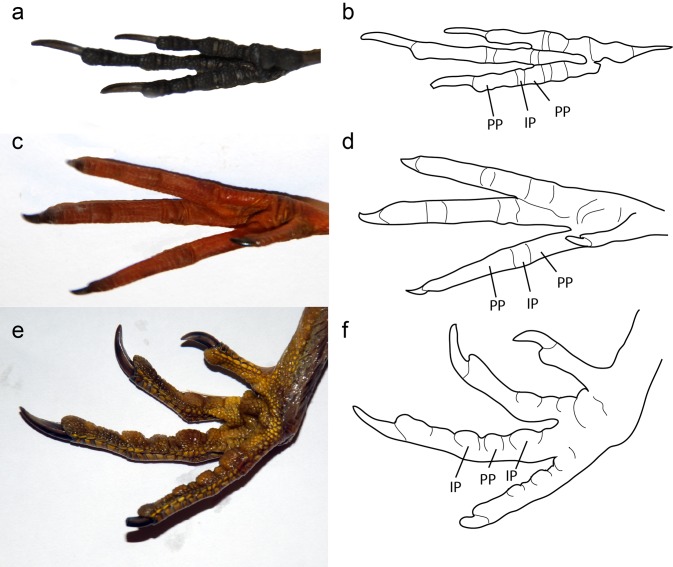
Examples of modern bird foot morphology. Photographs and line drawing of the feet of a passeriform (*Sitta carolinensis*), shorebird (*Tringa flavipes*), and ground-dwelling bird (*Tympanuchus cupido*) foot. (A) The foot of *S*. *carolinensis*, ventral view. This is an arboreal bird, and the large phalangeal pads are clearly defined. The smaller pads are the interphalangeal pads. (B) Line drawing of (A). PP = Phalangeal pad, IP = Interphalangeal pad. (C) Photograph of the foot *Tringa flavipes*, ventral view. Note that this is a water-marginal bird and the pads are much less clearly defined. (D) Line drawing of (C). (E) Photograph of the foot of *Tympanuchus cupido*, ventral view. (F) Line drawing of (E).

In modern birds, the foot morphology, especially that of the toe pads, varies strongly with respect to the life habit (Fig 8). Arboreal birds tend to have very large, fleshy, and expanded phalangeal pads, with strongly reduced interphalangeal pads over the joint (Fig 7), whereas birds that inhabit water-margin environments (e.g. lakeshores) tend to reduce the phalangeal pad and expand the interphalangeal pad while reducing overall fleshiness. Some ground-dwelling birds seem to retain the fleshiness of the pads, but also expand the interphalangeal pad at the expense of the phalangeal pad (Fig 8). Based on the soft tissues seen in specimen IVPP V13156, *Confuciusornis* possessed large, fleshy phalangeal pads, and the interphalangeal pads were small or absent, similar to modern arboreal perching birds. This is especially apparent on the left foot (Fig 4A), which displays concentrations of scales around the phalanx of the foot up to 3 mm wide, whereas the phalangeal joints have a much narrower area of scales associated with them. Concentrations of scales around the phalanxes are also visible on IVPP V13168, although they are more compacted (Fig 4C).

Although *Confuciusornis* did not possess a large bony keel to support the pectoralis muscle as in modern birds, and therefore was not capable of powerful flight for extended periods, it still had the same anatomical features that allow for flapping flight in modern birds save for the triosseal canal. These features include a sternal keel (although it is small and restricted, see [36]), and a large deltopectoral crest that would have allowed for larger deltoideus muscles for a more powerful upstroke. The pectoralis muscle also inserts on the ventral side of the deltopectoral crest [30]. This indicates that although *Confuciusornis* could not rotate the arm behind the body in the upstroke portion of the flight stroke, it could still conduct flapping flight on the downstroke and recovery portion of the upstroke. The thick, deep postpatagium on the fingers would provide a strong anchoring surface for the long, thick calami of the primary feathers. The robust and extensive propatagium would provide powerful lift in concert with the long primaries. Based on the evidence illuminated by laser fluorescence, *Confuciusornis* is unequivocally an arboreal bird with the capacity for short term (non-migratory) powered flight. If *Confuciusornis*, a primitive bird quite basal on the avian tree (e.g. see [39]), possessed a suite of characters so modern, it suggests that these features arose much earlier than perhaps previously implied. If that is so, then earlier rocks may contain the answers to many questions about the origin and early evolution of birds.

## References

[1] Martin LD, Zhou Z, Hou L, Fedducia A. *Confuciusornis sanctus* compared to *Archaeopteryx lithigraphica*. Naturwissenschaften. 1998; 85: 286–289.

[2] Zhang F, Hou L, Lian Q. Osteological microstructure of *Confuciusornis*: preliminary report. Vert Palasica. 1998; 35(2): 126–135.

[3] Chiappe LM, Ji S, Ji Q, Norell MA. Anatomy and systematics of the Confuciusornithidae (Theropoda: Aves) from the Late Mesozoic of northeastern China. Bull Amer Mus Nat Hist. 1999; 242: 1–89.

[4] Zhang Z, Gao C, Meng Q, Liu J, Hou L, Zheng G. Diversification in an Early Cretaceous avian genus: evidence from a new species of *Confuciusornis* from China. J Ornithol. 2009; 150: 783–790.

[5] Nudds RL, Dyke GJ. Narrow primary feather rachises in *Confuciusornis* and *Archaeopteryx* suggest poor flight ability. Science. 2010; 328: 887–889. 10.1126/science.1188895 20466930

[6] Wang X, Nudds RL, Dyke GJ. The primary feather lengths of early birds with respect to avian wing shape evolution. J Evol Bio. 2011; 24: 1226–1231. 21418115 10.1111/j.1420-9101.2011.02253.x

[7] O'Connor JK, Chiappe LM, Chuong C, Bottjer DJ, You H. Homology and potential cellular and molecular mechanisms for the development of unique feather morphologies in early birds. geosci. 2012; 2: 157–177. 10.3390/geosciences2030157 PMC3758748 24003379

[8] Paul GS. Comment on “Narrow Primary Feather Rachises in Confuciusornis and Archaeopteryx Suggest Poor Flight Ability”. Science. 2010; 330: 320. 10.1126/science.1192963 20947747

[9] Zheng X, Xu X, Zhou Z, Miao D, Zhang F. Comment on “Narrow Primary Feather Rachises in Confuciusornis and Archaeopteryx Suggest Poor Flight Ability”. Science. 2010; 330, 320. 10.1126/science.1193223 20947746

[10] Baumel JJ, Whitmer LM. Osteologia In: Baumel JJ, King AS, a JE, Evans HE, Vanden Berge JC editors. Hanbook of Avian Anatomy (2nd Ed.). Cambridge, MA, USA: Nuttal Ornithological Club; 1993 pp. 45–132.

[11] Brown RE, Cogley AC. Contributions of the propatagium to avian flight. J Exper Zool. 1996; 276: 112–124.

[12] Bachmann T, Emmerilich J, Baumgartner W, Schneider JA, Wagner H. Flexural stiffness of feather shafts: geometry rules of material properties. J Exper Bio. 2012; 215: 405–415. 22246249 10.1242/jeb.059451

[13] Homberger HD, Brush AH. Functional-morphological and biochemical correlations of the keratinized structures in the African Grey Parrot *Pisttacus erithacus* (Aves). Zoomorph. 1986; 106: 103–114.

[14] Brush AH. Convergent evolution of reticulate scales. J Exper Zool. 1985; 236: 303–308.

[15] Chalfie M, Tu Y, Euskirchen G, Ward WW, Prasher DC. Green fluorescent protein as a marker for gene expression. Science. 1994; 263(5148): 802–805. 8303295 10.1126/science.8303295

[16] Birkmann H, Lundin RF. Confocal microscopy: potential applications in micropaleontology. J Paleo. 1996; 70: 1084–1087.

[17] Schopf JW, Tripathi AB, Kudryavtsev AB. Three-dimensional confocal optical imagery of Precambrian microscopic organisms. Astrobio. 2006; 6: 1–16. 10.1089/ast.2006.6.1 16551223

[18] Schopf JW, Kudryavtsev AB. Confocal laser scanning microscopy and raman (and fluorescence) spectroscopic imagery of permineralized Cambrian and Neoproterozoic fossils In: Laflamme M, Schiffbauer JD, Dornbos SQ editors. Quantifying the evolution of early life Dordrecht, Netherlands: Springer Netherlands 2011; pp. 241–270.

[19] Kaye TG, Falk AR, Pittman M, Sereno PC, Martin LD, Burnham DA, et al Laser stimulated fluorescence in paleontology. PLoS ONE. 2015; 10(5), e0125923 10.1371/journal.pone.0125923 26016843 PMC4446324

[20] Pennycuick CJ. Modeling the Flying Bird. Burlington, MA, USA: Academic Press; 2008.

[21] Brown RE, Baumel JJ, Klemm RD. Anatomy of the Propatagium: The Great Horned Owl (*Bubo virginianus*). J Morph. 1994; 219: 205–224. 29865371 10.1002/jmor.1052190209

[22] Peters DS, Qiang J. Mugte *Confuciusornis* klettern? J Ornitho. (German). 1999; 140: 41–50.

[23] Perez-Tris J, Telleria JL, Age-related variation in wing shape of migratory and sedentary Blackcaps *Sylvia atricapilla*. J Avian Bio. 2001; 32(3): 207–213.

[24] Tucker VA. Gliding birds: Reduction of induced drag by wing tip slots between the primary feathers. J Exper Bio. 1993; 180: 285–310.

[25] Tucker VA. Drag reduction by wing tip slots in a gliding Harris' hawk, Parabuteo unicinctus. J Exper Bio. 1995; 198: 775–781. 9318544 10.1242/jeb.198.3.775

[26] Sachs G, Moelyadi MA. Effect of slotted wing tips on yawing moment characteristics. J Theor Bio. 2006; 239(1): 93–100. 16199060 10.1016/j.jtbi.2005.07.016

[27] Zhou Z, Barrett PM, Hilton J. An exceptionally preserved Lower Cretaceous ecosystem. Nature. 2003; 421: 807–814. 10.1038/nature01420 12594504

[28] Barrett PM, Hilton JM. The Jehol Biota (Lower Cretaceous, China): new discoveries and future prospects. Int Zool. 2006; 1: 15–17. 10.1111/j.1749-4877.2006.00006.x 21395985

[29] Savile DBO. Adaptive evolution in the avian wing. Evol. 1957; 11(2): 212–224.

[30] Baumel JJ, Whitmer LM. Myologia In: Baumel JJ, King AS, Breazile JE, Evans HE, Vanden Berge JC editors. Hanbook of Avian Anatomy (2nd Ed.). Cambridge, MA, USA: Nuttal Ornithological Club; 1993 pp. 189–247.

[31] Martin LD, Lim JD. Soft body impression of the hand in Archaeopteryx. Curr Sci. 2005; 89(7): 1089–1090.

[32] Brown RE, Baumel JJ, Klemm RD. Mechanics of the Avian Propatagium: Flexion-Extension Mechanism of the Avian Wing. J Morph. 1996; 225: 91–105. 10.1002/jmor.1052250108 29865338

[33] Larssen HCE, Wagner GP. Pentadactyl Ground State of the Avian Wing. J Exp Zool. 2002; 294: 146–151. 10.1002/jez.10153 12210115

[34] Zinoviev AV. An Attempt to Reconstruct the Lifestyle of Confuciusornithids (Aves, Confuciusornithiformes). Paleo J. 2009; 43(4): 444–452.

[35] Navalón G, Marugán-Lobón J, Chiappe LM, Sanz JL, Buscalioni AD. Soft-tissue and dermal arrangement in the wing of an Early Cretaceous bird: Implications for the evolution of avian flight. Sci Rep. 2015; 5, 14864 10.1038/srep14864 26440221 PMC4594305

[36] Zhou Z, Farlow J. Flight capability and habits of Confuciusornis. In: J Gauthier and LF Gall editors. New perspectives on the origin and early evolution of birds; proceedings of the International Symposium in honor of John. H Ostrom. New Haven, CN USA:Yale University Press. 2001; pp. 237–254.

[37] Proctor NS, Lynch PJ. Manual of Ornithology: Avian structure and function New Haven, CN, USA: Yale University Press; 1993.

[38] Burnham DA, Fedducia A, Martin LD, Falk AR. Tree climbing: a fundamental avian adaptation. J Sys Paleo. 2011; 9(1): 103–107.

[39] Zhou Z, Clarke J, Zhang F. Insight into diversity, body size and morphological evolution from the largest Early Cretaceous enantiornithine bird. J Anat. 2008; 212: 565–577. 10.1111/j.1469-7580.2008.00880.x 18397240 PMC2409080

